# Application of a diagnosis-based clinical decision guide in patients with neck pain

**DOI:** 10.1186/2045-709X-19-19

**Published:** 2011-08-27

**Authors:** Donald R Murphy, Eric L Hurwitz

**Affiliations:** 1 Rhode Island Spine Center, 600 Pawtucket Avenue, Pawtucket, RI 02860 USA; 2 Department of Health Services, Policy and Practice, Alpert Medical School of Brown University, Providence, RI USA; 3 Department of Research, New York Chiropractic College, Seneca Falls, NY USA; 4 Department of Public Health Sciences, John A. Burns School of Medicine, University of Hawaii at Mānoa, Hawaii USA

## Abstract

**Background:**

Neck pain (NP) is a common cause of disability. Accurate and efficacious methods of diagnosis and treatment have been elusive. A diagnosis-based clinical decision guide (DBCDG; previously referred to as a diagnosis-based clinical decision rule) has been proposed which attempts to provide the clinician with a systematic, evidence-based guide in applying the biopsychosocial model of care. The approach is based on three questions of diagnosis. The purpose of this study is to present the prevalence of findings using the DBCDG in consecutive patients with NP.

**Methods:**

Demographic, diagnostic and baseline outcome measure data were gathered on a cohort of NP patients examined by one of three examiners trained in the application of the DBCDG.

**Results:**

Data were gathered on 95 patients. Signs of visceral disease or potentially serious illness were found in 1%. Centralization signs were found in 27%, segmental pain provocation signs were found in 69% and radicular signs were found in 19%. Clinically relevant myofascial signs were found in 22%. Dynamic instability was found in 40%, oculomotor dysfunction in 11.6%, fear beliefs in 31.6%, central pain hypersensitivity in 4%, passive coping in 5% and depression in 2%.

**Conclusion:**

The DBCDG can be applied in a busy private practice environment. Further studies are needed to investigate clinically relevant means to identify central pain hypersensitivity, oculomotor dysfunction, poor coping and depression, correlations and patterns among the diagnostic components of the DBCDG as well as inter-examiner reliability, validity and efficacy of treatment based on the DBCDG.

## Background

Neck pain (NP), along with related disorders such as cervical radiculopathy and headache, is very common. It is estimated that 30-50% of adults will experience some form of significant NP in any given year [1]. Further, work limitation due to NP occurs in 11-14% of individuals each year [2]. The recent Bone and Joint Decade Neck Pain Task Force identified the need for research that examines the clinical criteria for diagnosis as well as the best forms of treatment for patients with NP and related disorders [3]. Also recognized by the Neck Pain Task Force is the importance of applying a patient-focused approach that considers the biopsychosocial nature of NP [4,5].

Practice-based research that generates data in a "real world" environment has recently been emphasized as a useful tool for conducting comparative effectiveness research [6,7]. This movement calls for greater participation of private practice environments in clinical research [7].

A diagnosis-based clinical decision Guide (DBCDG) has been proposed for the purpose of guiding clinicians in the application of the biopsychosocial model in patients with NP. This has been referred to in previous publications as a diagnosis-based clinical decision rule. The approach attempts to identify specific characteristics in each individual patient from which treatment decisions can be made [8]. It is influenced by the existing disparate literature on the diagnosis and management of patients with spinal pain [9]. Initial observational cohort studies have suggested that the outcomes of treatment based on the DBCDG may be promising [10-13], however further study is needed to determine the generalizability of these findings as well as whether they can be replicated in controlled studies.

This study is part of a larger research effort to investigate the clinical utility of the DBCDG. This effort began with observational cohort studies in defined populations that documented the clinical outcomes of patients with cervical radiculopathy [10], lumbar spine stenosis [11], pregnancy-related lumbopelvic pain [12] and lumbar radiculopathy secondary to disc herniation [13]. These were practice-based observational studies without randomization and control. Future studies will require identifying specific subgroups of patients that have certain multifactorial diagnoses according to the answers to the three questions of diagnosis. Given the fact that there is a variety of potential factors that can contribute to the experience of NP, there could potentially be a large number of different diagnoses, making subgrouping difficult. However, clinical experience seems to suggest that there are enough commonalities among NP patients that the actual number of different diagnostic factors is small enough to make subgrouping possible. The purpose of this study is to identify the frequency with which clinicians trained in the application of the DBCDG identify the individual findings in order to inform future studies of this approach.

## Methods

The study protocol was approved by the Institutional Review Board of New York Chiropractic College. It was also reviewed by the Health Insurance Portability and Accountability Act (HIPAA) compliance officer of the facility at which the data were gathered and was deemed to be in compliance with HIPAA regulations. All subjects signed informed consent forms, agreeing to have their data included in the study.

Cross-sectional data were gathered on consecutive patients seen at the Rhode Island Spine Center between 2/7/08 and 2/26/09.

### Participants

Patients were included in the study if they 1) had NP with or without associated head or upper extremity pain; 2) were age 18 years or older; 3) provided informed consent; 4) were able to communicate well in English; 5) had a Bournemouth Disability Questionnaire (BDQ) score of 15 or higher.

### Clinical Examination

All examinations were carried out by one of two chiropractic physicians, one with over 20 years experience and the other with nine years experience, or by a physical therapist with over 10 years experience. All had a minimum of 50 hours of postgraduate training in the McKenzie method. The physical therapist also had 80 hours of postgraduate training in manual therapy. Several discussions between the examiners took place over the course of five years prior to commencing data gathering on the application of the DBCDG. This occurred in the form of monthly clinical meetings in which the application of the DBCDG in particular patients was discussed as well as recent developments in the literature related to the evaluation and management of patients with NP. History and examination were performed according to the usual course of patient care at the Rhode Island Spine Center. These data, along with patient demographic data, and data from standardized outcome measurement instruments were then entered on a spreadsheet by a chiropractic intern. The standardized outcome measurement instruments were those tools used in the normal course of patient care at the facility at which the study was conducted to measure improvement in pain and perceived disability. These instruments were the Bournemouth Disability Questionnaire (BDQ) [14,15] and Numerical Rating Scale [16] for pain intensity.

Details of the proposed DBCDG are published elsewhere [8,9] but the approach is based on three questions of diagnosis:

1. Are the symptoms with which the patient is presenting reflective of a visceral disorder or a serious or potentially life-threatening disease?

The purpose of this question is to identify signs and symptoms suggestive of non-musculoskeletal problems for which NP may be among the initial symptoms. Gastrointestinal and anterior neck disorders are included in addition to such "red flag" disorders as fracture, infection and malignancy.

2. From where is the patient's pain arising?

In the majority of cases it is not possible to know with absolute certainty what the pain generating tissue is. However there is evidence that characteristics of the pain generating tissue can be reliably identified [17-24] and that treatment decisions can be made based on these characteristics [10,24].

3. What has gone wrong with this person as a whole that would cause the pain experience to develop and persist?

With this question the clinician seeks to identify factors that serve to perpetuate the ongoing pain and suffering experience. These factors may be somatic, neurophysiological or psychological. Often more than one perpetuating factor is identified.

**Figure 1:**
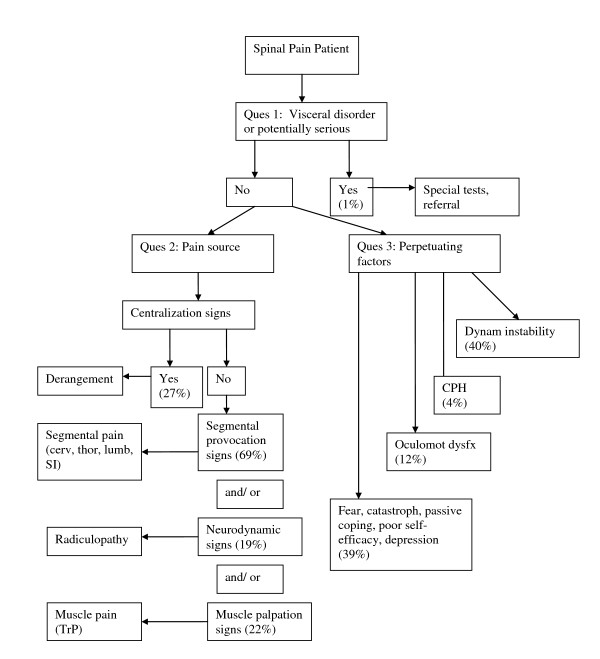
**Diagnostic algorithm for the application of the DBCDG**. Adapted with permission from: Murphy DR, Hurwitz EL. A theoretical model for the development of a diagnosis-based clinical decision rule for the management of patients with spinal pain. BMC Musculoskelet Disord 2007;8:75. cerv = cervical; thor = thoracic; lumb = lumbar; SI = sacroiliac; TrP = trigger point; CPH = central pain hypersensitivity; dysfx = dysfunction.

**Figure 2:**
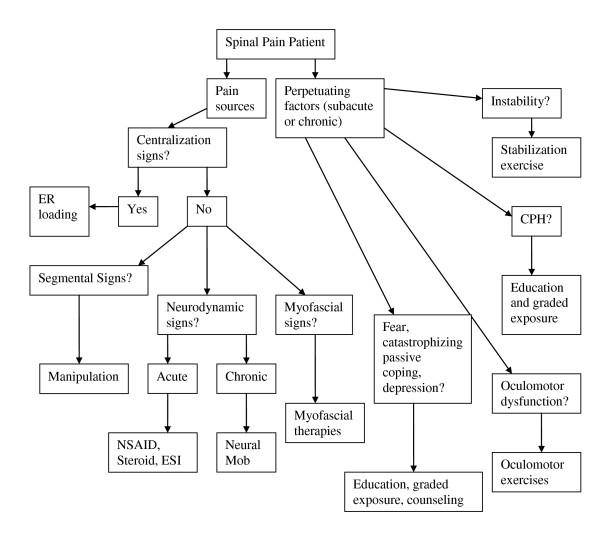
**Management algorithm for the application of the DBCDG**. Reprinted with permission from: Murphy DR, Hurwitz EL. A theoretical model for the development of a diagnosis-based clinical decision rule for the management of patients with spinal pain. BMC Musculoskelet Disord 2007;8:75. ER = end range; NSAID = non-steroidal anti-inflammatory drugs; ESI = epidural steroid injection; mob = mobilization; CPH = central pain hypersensitivity.

Following each new patient encounter the answers to the three questions of diagnosis were documented on a standardized form (see Additional File 1). The combined answers to the three questions of diagnosis are formulated into a working diagnosis (Figure 1) from which a management strategy is derived (Figure 2). In many cases, the working diagnosis is multifactorial, leading to a multi-modal management strategy.

In seeking an answer to the first question of diagnosis, standard history and examination procedures were used. In cases in which it was warranted, such as the presence of red flags for fracture, dislocation, infection or malignancy, profound motor loss, or signs and symptoms reflective of visceral disease, special tests such as radiographs, MRI or blood tests were ordered [25,26].

In seeking the answer to the second question of diagnosis, four signs are considered:

a. Centralization signs, detected via the McKenzie end-range loading examination. Details of this examination can be found elsewhere [27] however the approach involves moving the cervical spine, either by patient- or examiner-generated maneuvers, to the end of the range of motion in various directions. A typical centralization sign is detected if movement in a certain direction causes progressive "centralization" of the patient's symptoms, i.e., movement of the symptoms from the periphery (upper extremity and/or scapula) to the axial spine. Also considered a centralization sign would be a progressive decrease in pain intensity even if movement of the pain to the center was not perceived.

b. Segmental pain provocation signs, detected via segmental palpation as described by Jull, et al [19,28]. This involved the patient lying prone and the examiner using the hands to move the overlying tissues lateral to medial and applying pressure as close to zygapophyseal joint as possible. The presence of segmental pain provocation signs was based on the examiner perceiving increased resistance to this pressure relative to other segments and the patient reporting reproduction of the NP [19,28]. In cases in which there was a discrepancy between the amount of resistance perceived by the examiner and reproduction of pain perceived by the patient, pain reproduction was given priority.

c. Neurodynamic signs, detected by tests designed to compress, decompress or stretch the cervical nerve roots [22,29]. The cluster of tests that formed the core of this examination was a) the brachial plexus tension test, in which stretch is applied to the neural structures of the cervical spine and upper extremity to determine whether this reproduces the patient's pain (with localizing and sensitizing maneuvers applied for confirmation) [30]; b) rotation to the side of symptoms being limited due to reproduction of pain; c) the cervical distraction test in which the head of the seated patient is move superiorward to distract the cervical spine and the patient is asked if this relieves pain, and d) the maximum cervical compression test in which the cervical spine of the seated patient is moved into lateral flexion toward the side of symptoms and slightly extended and pressure is applied to the top of the head to determine if this reproduces the patient's pain. Wainner, et al [22] found that the presence of positive findings on three of these tests indicated at least a 65% probability of the presence of cervical radiculopathy. The presence of positive findings in all four tests increased the probability to 90%. This was reinforced by neurologic examination looking for nerve root-specific neurologic deficit although neurologic deficit was not necessary for the determination of the presence of neurodynamic signs.

d. Myofascial signs, detected by palpation [20,23,31,32] in which the examiner searches for a taut band within a muscle and a nodular formation within the taut band (a trigger point). Pressure is applied to the nodule to determine if this reproduces the patient's pain. Trigger points can occur in latent form in individuals without pain and as such it is considered important to not only identify the presence of a trigger point but to determine whether it is diagnostically relevant in any given patient [33]. Thus, these signs were only recorded if the clinician felt they were diagnostically relevant to the patient's NP.

In seeking answers to the third question of diagnosis, four factors were considered [8]:

1. Dynamic instability, detected through clinical tests of motor control for the cervical spine [34-37]. Impairment of the motor control system has been theorized to lead to perpetuation of pain and disability as a result of ongoing microtrauma to the tissues of the spine [38-40]. The primary test used for this purpose was the cervical stability test in which the head of the supine patient is held with the upper cervical spine slightly flexed and it is determined if the patient can maintain this position when the examiner lets go of the head [35-37,41].

2. Oculomotor dysfunction. This is commonly associated with pain that occurs after cervical trauma in patients who experience delayed recovery [42-44]. There is some evidence of a correlation between oculomotor dysfunction and findings on tests of head repositioning [45] however the sensitivity and specificity are not very high [46]. Other clinical tests have been proposed [47] but these have not been assessed for reliability and validity. Thus, there is currently no clinical examination procedure that has been shown to have high clinical utility in detecting oculomotor dysfunction. However as oculomotor exercises have been shown to be effective [48,49] it was felt that a decision-making criterion was needed by which to determine which patients should at least be suspected of potentially having oculomotor dysfunction. As oculomotor dysfunction has been associated with cervical trauma, this factor was recorded as positive in any patient whose NP arose from trauma.

3. Central pain hypersensitivity (CPH), detected through observation of pain behavior in response to stimuli as well as through cervical nonorganic signs [50]. This was based on the findings of Fishbain, et al [51] who reviewed the literature on nonorganic signs in patients with low back pain and found that these signs, in addition to predicting poor functional abilities and poor outcome to treatment, were associated with greater pain levels and that the majority of these signs can be explained on the basis of pain intensity. Intensity of chronic pain is thought to reflect central nervous system processes (termed here central pain hypersensitivity) in addition to peripheral processes [52]. Because of this, these signs were only used in chronic NP patients and not in acute NP patients. However, the sensitivity and specificity of the use of nonorganic signs for suspected CPH is not known.

4. Psychological factors such as fear [53], catastrophizing [54], passive coping [55], depression [56] and poor self-efficacy [57]. There is evidence that at least some of these factors co-exist in individual patients [57-60] and that while it is likely best to measure more than one factor, it is not necessary to detect all of them in order to identify a significant psychological component to the clinical picture [61]. Based on this, and consistent with the need to obtain quality information in the context of a busy clinical environment with minimal burden to the patient, measurement of all these factors, which would have necessitated each patient completing multiple questionnaires, was not undertaken. Three measures were used for the purpose of identifying fear beliefs, coping strategies and depression. Fear beliefs were measured using the 11-item Tampa Scale for Kinesiophobia (TSK) [62]. A score of 27 was considered indicative of clinically meaningful fear beliefs. This number was adapted from Vlaeyen, et al [63] who used a cutoff score 40 using a previous 17-item version of the TSK and Woby (personal communication 3 August, 2009) whose unpublished data suggested a score of 26 to 27 to be associated with clinically meaningful fear beliefs. In addition, two questions from the Coping Strategies Questionnaire [64] which have previously been found to be predictive of changes in disability in LBP patients [65] were used to measure patients' perception of their control over the pain. At the time of data collection, no data were available regarding whether a particular score with these questions constitutes a threshold for clinically meaningful difficulty with coping strategies, i.e., that score that represents a reasonable cutoff between the presence or absence of coping strategies that may perpetuate ongoing pain, suffering and disability. The depression subscale of the BDQ [15] was used to measure depression. In the development of the BDQ, the question related to depression was found to correlate well with the Zung Depression scale [14] and the Mental Health scale of the SF36 instrument [15]. As with the coping strategies questions, no data are available by which to determine a threshold for clinically meaningful depression with this question.

Patients also completed the BDQ [15] and the Numerical Rating Scale for pain intensity (NRS) [16].

## Statistical analysis

Descriptive statistics were used to characterize the study population. Frequencies, percentages, and 95% confidence intervals were computed for categorical variables; means, standard deviations, medians, and ranges were computed for continuous variables. Data management and statistical analyses were conducted with Microsoft Excel and SAS (version 9.1, Cary, NC).

## Results

Data were gathered on 95 patients, 63% of whom were female. No patient declined participation. Baseline characteristics are presented in Table 1.

**Table 1:** Baseline characteristics

Variable	Mean (SD)	Median	Interquartile range	Range
Age	45.0 (14.0)	43.5	18.0	19-79

Neck Pain Duration (days)	881.7 (2166.3)	122.0	709.0	1 day to 13 years

Disability	40.6 (14.4)	40.0	25.0	15-67

Pain	6.8 (1.9)	7.0	2.0	2-10

Fear	24.6 (5.8)	25.0	6.0	11-42

Coping	5.1 (2.3)	6.0	2.5	0-10

Depression	4.7 (3.1)	5.0	5.0	0-10

Disability was measured using the Bournemouth Disability Questionnaire; Pain was measured using the Numerical Rating Scale); Fear was measured using the Tampa Scale for Kinesiophobia; Coping was measured using a 2-item coping screen; Depression was measured using item #5 on the Bournemouth Disability Questionnaire

Regarding the first question of diagnosis, one patient (1%) was positive. This was a 77-year-old man with recent onset neck pain and temporal headache and marked tenderness over the temporal artery who was referred for blood tests to rule out temporal arteritis. Data regarding the second and third questions of diagnosis are provided in tables 2 and 3, respectively. Displayed are the percentage of patients in whom each sign was identified and the 95% confidence intervals for each. The most common finding under the second question of diagnosis was segmental pain provocation (69%; 95% CI = 59.8-78.5) and under the third question of diagnosis was dynamic instability (40%; 95% CI = 30.2-49.9).

**Table 2:** Responses to the second question of diagnosis

Diagnostic sign	Frequency	Percent (95% CI)
Centralization sign	26	27.4 (18.4-36.3)

Segmental pain provocation Sign	65	69.2 (59.8-78.5)

Neurodynamic sign	18	19.0 (11.1-26.8)

Myofascial sign	21	22.1 (13.8-30.5)

"From where is the patient's pain arising?".

CI = confidence interval

**Table 3:** Responses to the third question of diagnosis, "What has gone wrong with this person as a whole that would cause the pain experience to develop and persist?"

Diagnostic sign	Frequency	Percent (95% CI)
Dynamic instability	38	40.0 (30.2-49.9)

Oculomotor Dysfunction	11	11.6 (5.1-18.0)

Central pain hypersensitivity	4	4.2 (0.2-8.3)

Fear	30	31.6 (22.2-40.9)

Passive coping	5	5.3 (0.8-9.8)

Depression	2	2.1 (0-5.0)

CI = confidence interval

## Discussion

Identifying specific diagnostic characteristics in patients with NP upon which treatment decisions can be made has been established as a research priority [3]. This is challenging as 1) NP is multifactorial; 2) the factors that contribute to the suffering of NP patients involve somatic, neurophysiologic and psychological processes, and 3) most of the factors that contribute to this suffering cannot consistently be unequivocally identified using objective tests. Thus, NP is very much a clinical diagnosis. The DBCDG has been proposed in an attempt to assist clinicians in responding to these challenges. Further research is needed to determine the usefulness of this approach.

In addition there is a great need for research that documents the clinical processes and outcomes that occur in the "real-world" environment of clinical practice as a contributor to comparative effectiveness research [6,7]. This study was part of a broad research strategy to respond to the need for practice-based research by investigating the clinical utility of the DBCDG for patients with NP. Its purpose was to document the prevalence of the clinical findings in NP patients evaluated according to the DBCDG. Future studies are planned that will investigate correlations and patterns among the diagnostic components and investigate the reliability and efficacy of this approach in patients with NP. Preliminary data suggests that outcomes in select patients groups may be favorable [10-13,10,11,66,67], but this is based on observational studies without randomization or control. High level studies will be required to further investigate the clinical utility of the DBCDG in NP patients. Conducting further studies will require subgrouping patients according to diagnosis. In order to create subgroups it must first be determined how many different possible diagnoses are found when utilizing the DBCDG. This study was the first step in this process.

Segmental pain provocation signs were the most frequent finding under the second question of diagnosis with a prevalence of 69%. These signs were originally thought to reflect zygapophyseal joint pain [18] although recent evidence argues against this [68]. The prevalence of this finding is higher than the estimated prevalence of zygapophyseal joint pain of 50% in patients with chronic neck pain or headache [69-71]. This difference may be due to the mix of acute and chronic patients in the present cohort or may reflect the possibility that segmental pain provocation signs may provoke pain arising from other structures in addition to those related to the zygapophyseal joints. Further work is needed to determine from what tissues the pain elicited with these signs is arising.

Centralization signs were found in 27.4% of patients. No data is found in the literature on the prevalence of this finding however the prevalence found here is substantially lower than the 45-50% prevalence of centralization in back pain patients [72-74]. Data were only gathered at the initial visit. However the usual clinical protocol at the clinic at which this study was performed calls for the determination of the centralization response to occur over the course of several visits as this has been shown to be more accurate, at least in patients with low back pain [75]. Thus, the percentage of patients who were centralizers may be underestimated here. On the other hand, as the prevalence of this finding is unknown, it is also possible that the percentage of centralizers may be overestimated in this study.

Radicular signs were found in 20% of patients. While the incidence of cervical radiculopathy in the general population has been found to be 83.2 per 100,000 population [76], no data are found in the literature regarding the prevalence of this diagnosis among NP patients. However, this is similar to the 24% prevalence reported in a cohort of low back pain patients evaluated using the DBCDG [77]. The prevalence of myofascial signs of 22% was more than double that found in a cohort of low back pain patients evaluated using the DBCDG [77]. It is not clear whether this reflects a higher prevalence of this finding in NP patients in comparison to back pain patients or to the fact that the reliability of muscle palpation signs has been found to be greater in the cervical spine [20,23,31] than the lumbar spine [78-80].

There were three factors under the third question of diagnosis for which the prevalence was quite low. Only 4% of patients were identified to have central pain hypersensitivity, only 5% were identified to have passive coping and only 2% were found to have depression. As these factors have been found to be significant in the development of chronic NP [55,56,81], it is likely that the low prevalence of the diagnosis of these factors in this study represents under-recognition. Another possibility is that this cohort did not display these features or that sampling error led to low prevalence. It also may be that the means used in this study to identify these factors were suboptimal. In the case of central pain hypersensitivity, there is no well established means of identification. Further work on the development of non-organic signs in the cervical spine may improve the identification of these signs [82]. In addition, there may be other methods, such as pressure algometry [83], that may be useful in the detection of central pain hypersensitivity. In the case of passive coping and depression, the scales used to identify these factors have no established threshold score that identifies the presence of clinically meaningful problematic coping strategies and depression. The mean score on the coping strategies questions was 5.1 out of a possible 12 and on the depression subscale on the BDQ was 4.6. A recent study found that a baseline coping score of less than 8 had the highest sensitivity and score of less than 4 had the greatest specificity in identifying a NP patient who is not likely to experience clinically meaningful improvement in pain and disability [84]. These data will be used as the basis for further investigation that attempts to establish these thresholds. It is expected that this will increase clinical utility of these questions in identifying the patient who has problematic coping strategies and depression.

In this study only fear, coping and depression were measured. Other important psychological factors that are of importance in patients with NP, such as catastrophizing and poor self-efficacy, were not specifically measured. There is some evidence that these various psychological factors interact, rather than occurring in isolation [57-60] and that identification of more than one factor, but not necessarily all factors is adequate [61]. As this was a practice-based research project that is part of the investigation of identification of key elements in the perpetuation of NP in a "real-world" environment, it was decided that fear, coping and depression would be measured rather than attempting to measure all potentially relevant factors. Further work is needed to determine whether this is a worthwhile approach for clinicians.

This study had several limitations. First, the sample size of 95 patients was small. In addition, all data were gathered at a single clinic and thus it is not known whether the information is generalizable. Also the design was observational and the practitioners were not blinded to the findings on each patient. The suspicion of the presences of oculomotor dysfunction was made based on a traumatic onset of the NP. It is not known whether all patients whose NP is caused by trauma have oculomotor dysfunction or what percentage, if any, of patients with non-traumatic neck pain have this condition. The decision to use trauma as the criterion in this case was based on the common association found in the literature between oculomotor dysfunction and cervical trauma and the absence (thus far) of a diagnostic test that identifies this condition and that has utility in a busy clinic environment. The approach to oculomotor dysfunction may be revised based on the evolving evidence regarding clinical tests of oculomotor reflexes [47]. Finally, because this was a pragmatic study in which data were gathered during the normal course of clinical care detailed information regarding psychological factors was not obtained as this would have required patients filling out several questionnaires. On the other hand, the fact that this study was carried out in a real-world environment may also be a strength in that it suggests that the information applies to the environment in which patients are most commonly cared for as opposed to the controlled environment of a research center.

## Conclusion

The DBCDG can be applied in a busy private practice setting. It appears possible to investigate the usefulness of the DBCDG through practice-based comparative effectiveness research. Further research is needed to investigate the validity of the questions used in this study to identify problematic coping strategies and depression as well as to establish a threshold for a "positive" and "negative" finding for these measures. In addition, there is need to find better clinical means of identifying central pain hypersensitivity. Research is also needed to investigate correlations and patterns among the individual components of the approach, the reliability and validity of the diagnoses and the clinical utility and efficacy.

## Competing interests

The authors declare that they have no competing interests.

## Authors' contributions

DRM originally conceived of the study served as an examiner. He was also the main writer of the manuscript. ELH was responsible for statistical analysis and writing and editing the manuscript. Both authors read and approved the final manuscript.

## Supplementary Material

Additional file 1 **Standardized form on which the answers to the three questions of diagnosis were documented**. Click here for file
