# Supplementary material for: Application of a diagnosis-based clinical decision guide in patients with neck pain
Source: Chiropr Man Therap. 2011 Aug 27;19:19. doi: 10.1186/2045-709X-19-19 (PMC3177766; doi:10.1186/2045-709X-19-19)
Supplement: Additional file 1 — Standardized form on which the answers to the three questions of diagnosis were documented. [file 2045-709X-19-19-S1.PDF]

Name________________________________ Date____________________

Doctor's Diagnosis Form

Question 1. Visceral/ redflag

Question 2. Pain source

Centralization: Cervical____ Lumbar____ DP________

Segmental: Cervical____ Lumbar____ SI____

Neurodynamic: Cervical____ Lumbar_____ Central_____

Muscle palpation: Cervical____ Lumbar_____ Pelvic_____

Question 3. Perpetuating factor

Instability: Cervical_____ Lumbar – hip ext_____ Lumbar – Seg Inst_____

Pelvic (ASLR) _____

CPH:_____

Fear:_____

Passive coping:_______

Depression:_________

Dr.'s initials _________
